# Comparative Assessment of Group Contribution Models for Estimating the Isobaric Heat Capacity of Ionic Liquids

**DOI:** 10.1021/acsomega.6c06472

**Published:** 2026-10-13

**Authors:** Peterson Y. G. de Medeiros Araújo, Nathan S. Evangelista, Frederico R. do Carmo, Filipe X. Feitosa, Hosiberto B. de Sant’Ana

**Affiliations:** † Grupo de Pesquisa em Termofluidodinâmica Aplicada, Departamento de Engenharia Química, 28121 Universidade Federal do Ceará, Campus do Pici, Bloco 709, Fortaleza, State of Ceará 60455-760, Brazil; ‡ Departamento de Engenharia Química e de Materiais, 474972 Universidade Federal de Lavras, Campus Universitário, 37200-900 Lavras, MG, Brazil; § Núcleo de Pesquisa em Economia de Baixo Carbono, Departamento de Engenharia e Tecnologia, Universidade Federal Rural do Semi-Árido, Campus Leste., 59625-900 Mossoró, Rio Grande do Norte, Brazil

## Abstract

Eight widely used group-contribution models were evaluated for their ability to estimate the isobaric heat capacity (*C*_p_) of ionic liquids (ILs). A curated database containing 9037 experimental data points for 142 ILs, covering eight cation families, temperatures from 190 to 524.87 K, and heat capacities from 93.0 to 1805.7 J·mol^–1^·K^–1^, was assembled to assess model performance under diverse conditions. Three main evaluation criteria were applied: predictive accuracy, functional-group flexibility, and ability to represent structural diversity of cation–anion combinations. Among the evaluated models, “GAR”, “SOR”, and “MIA” exhibited the best predictive accuracy, with overall % AARD values of 5.10%, 4.30%, and 4.22%, respectively. The “MIA” model is particularly suitable for imidazolium- and pyridinium-based ILs, relevant in industrial contexts. The “SAT” model also proved promising for CAMD applications due to its flexible atomic-contribution framework. Overall, the study offers practical guidance for selecting group-contribution methods when experimental data are unavailable.

## Graphical abstract

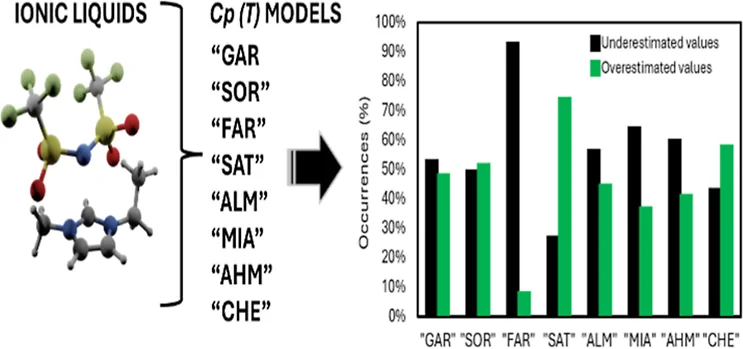

## 1 Introduction

Ionic liquids (ILs) are typically defined as salts composed entirely of cations and anions that remain in the liquid state under ambient or near ambient conditions. These liquids are also termed room-temperature molten salts, liquid organic salts, or fused salts.^1^

Over the past decades, ILs have emerged as a promising class of materials,^2^ attracting considerable research interest due to their intrinsic physical and chemical properties, such as low flammability,^3^ negligible vapor pressure,^4^ the ability to dissolve polar and nonpolar compounds,^5^ and a wide electrochemical window.^6^ These properties arise from the structural diversity of ILs, as they are composed of a wide variety of organic cations combined with organic and inorganic anions.^7,8^ As a result, studies on their structural and functional properties have become increasingly important in both academic and industrial contexts, especially regarding temperature-dependent properties such as density, viscosity, speed of sound, and isobaric heat capacity.^9–11^

The isobaric heat capacity (*C*_p_) is a key property in industrial process design,^12^ especially for solvents. It enables energy balance calculations in chemical engineering because it is required to compute enthalpy variations in process streams, provides information on phase transitions, and characterizes a compound’s thermodynamic behavior. The isobaric heat capacity of ILs is of particular interest in developing energy storage systems because of their high density and thermal capacity.^13^ This property also plays a critical role in chemical equilibrium modeling and thermodynamic analyses.

Given the vast number of possible cation–anion combinations that can form an ionic liquid, obtaining experimental *C*_p_ data for all conceivable ILs is a significant challenge. This task is demanding, time-consuming, and costly, especially when experiments must cover wide temperature (*T*) ranges, as is often the case in scientific investigations.^12^ One practical approach is to apply estimation models to provide *C*_p_ vs *T* behavior with acceptable accuracy for engineering calculations.^14^

The Group Contribution (GC) concept has become a practical and widely used framework for estimating thermodynamic properties, including isobaric heat capacity.^12^ Its central idea is that the substance’s structure is divided into functional groups, whose individual contributions are combined according to additive rules to calculate the desired property.^14^ The favorable balance between simplicity and accuracy offered by the GC approach has motivated the development of several models for predicting the isobaric heat capacity of ILs over the past two decades.

After an extensive literature survey, we identified various GC models^15–22^ capable of estimating the isobaric heat capacity of ILs at different temperatures. Although the results reported in the original papers are promising, the strengths and limitations of each model must be carefully examined to support proper model selection for practical applications. For instance, some models may perform well for specific cation families but yield less reliable predictions for others. In some cases, the set of functional groups in a model may be insufficient to describe standard structural units (e.g., alkyl chains) that occur in commercial ILs. Moreover, a model’s accuracy depends directly on the quality of the experimental *C*_p_ data used in parameter fitting. In other words, *C*_p_ data obtained from impure IL samples may contain errors that can propagate to the fitted parameters and ultimately affect model performance.

In this work, eight group contribution models (GCMs) were selected for quantitative evaluation: Gardas and Coutinho (2008),^15^ Soriano et al. (2010),^16^ Farahani et al. (2013),^22^ Sattari et al. (2014),^21^ Albert and Müller (2014),^18^ Müller and Albert (2014),^17^ Ahmadi et al. (2015),^20^ and Chen et al. (2019).^19^ These models constitute the core set of established GCMs for ionic liquid heat capacities, covering the main conceptual frameworks proposed in the literature over the past decades, including cation-based, anion-based, and atom-based formulations.

Considering this scenario, we conducted a systematic comparison of the GC models reported in the literature for predicting the isobaric heat capacities of ILs. To carry out our analyses, we compiled a comprehensive experimental database covering a wide range of temperatures and a diverse set of cations and anions present in the commercially available ILs. Comparing all models using the same database is a key aspect of our study, as it allows a consistent and unbiased evaluation of their performance. The models were analyzed using various criteria, including predictive accuracy, flexibility of the functional groups, and their ability to represent the wide variety of cations and anions in our database. Our goal is to provide clear guidance on the applicability and limitations of each model for future use in the absence of experimental data. To the best of our knowledge, no previous study has applied a methodology comparable to ours, which justifies the importance of this work.

## 2 Experimental Data Bank

Reliable evaluation of estimation methods requires high-quality experimental data, especially for properties such as the isobaric heat capacity.^23^ For this reason, the experimental values used in this study were carefully selected with attention to the reported sample purity and the rigor of the experimental protocols reported in the original publications. Heat capacity data for ILs may vary significantly because of sample impurities, calibration inconsistencies, or limitations inherent in the measurement apparatus. Studies employing advanced calorimetric techniques, including adiabatic calorimetry, have demonstrated that uncertainties as low as 1–5% can be achieved for highly pure samples,^24,25^ whereas larger uncertainties generally reflect experimental anomalies.

As a basis for all analyses, we initially compiled a comprehensive database from the NIST databank.^26^ It consisted of 9037 experimental *C*_p_ data points for 142 ILs within a temperature interval of 190.00 to 524.87 K. To examine the effect of data quality on model performance, two scenarios were considered for analysis: (i) using the entire database and (ii) using a refined subset containing only measurements with reported experimental uncertainties ≤10%.

To ensure consistency in assessing data quality, all uncertainty values extracted from the NIST Ionic Liquid Database^26^ correspond to the combined expanded uncertainties reported at a 95% confidence level. The uncertainties are evaluated within the framework of the IUPAC project on ionic liquids and are consistent with the principles of the Guide to the Expression of Uncertainty in Measurement (GUM).^27,28^ In this context, NIST reviews the uncertainty information provided by the original authors and, when necessary, applies standardized assumptions to address common issues related to synthesis, purification, analytical procedures, and measurement techniques in ionic liquid research. As a result, the reported uncertainty values are standardized across data sets, considered preliminary, and may be updated as additional experimental details or improved assessments become available. This GUM-based standardization ensures that the uncertainty values used in this work are directly comparable and represent a reliable current assessment of the measurement quality.

The group contribution models assessed in this work are fixed-parameter correlations, and no refitting or regression was performed using the present database. For this reason, statistical procedures such as cross-validation or external test set partitioning are not applicable here. Moreover, applying such procedures would not be consistent with evaluating all published models under identical, comprehensive experimental conditions using a common database rather than model-specific training and testing subsets.

In addition, the database used in this study, extracted from the NIST Ionic Liquids Database,^26^ was compiled without distinguishing whether specific data points had been previously used to parametrize the evaluated group contribution models. Because the original training data sets are not always fully reported or readily traceable in the literature, we could not rigorously separate calibration from external validation data. Consequently, some degree of overlap between the present database and the data sets used during model development cannot be completely excluded. Therefore, the reported performance statistics should be interpreted as indicators of the overall applicability and accuracy of the models for ionic liquids included in the compiled database rather than as a strict assessment of their predictive capability based exclusively on independent external data.

The refined subset included 4118 data points across 62 ILs, all within the same temperature range as the whole database. The adoption of a ≤10% uncertainty threshold aligns with established experimental standards^29,30^ in the field and provides a conservative yet inclusive criterion, ensuring the reliability of the refined data set while still capturing a broad range of chemical diversity.

Although high-pressure heat capacity data for ILs are available in the NIST database,^26^ they were not included in our analysis. The models examined in this study are exclusively temperature-dependent; therefore, applying them to predict *C*_p_ values for the same IL at the same temperature but at different pressures would yield identical results. Furthermore, because the models apply solely to liquids, we excluded heat capacity data for ILs in solid and metastable liquid states, retaining only *C*_p_ measurements for ILs in the stable liquid state. Full details of the compiled database, including temperature, heat capacity, measurement apparatus, sample purity, supplier, and corresponding literature references, are provided in the Supporting Information. Also, a detailed summary of the minimum, maximum, and average % ARD and % AARD values for each ionic liquid and each model is provided in the Supporting Information (Table Min. and Max.), which helps readers better understand how extreme deviations influence the results and how sensitive the selected error metrics are to these values.

## 3 Group Contribution Methods

Table 1 provides a comprehensive overview of the estimation methods considered in this work.

**Table 1:** Selected Group Contribution Models for Isobaric Heat Capacity Estimation

Model	Acronym	Equation	Number of Cation Groups	Number of Anion Groups	Number of Side Groups	*T* range (K)^a^	*C*_p_ range (J·mol^–1^·K^–1^)
Gardas–Coutinho^15^	“GAR”	$C_{p}\left(J\cdot {mol}^{-1}\cdot K^{-1}\right)=R\left[A+B\left(\frac{T}{100}\right)+D{\left(\frac{T}{100}\right)}^{2}\right]$	5	27	3	196.36–663.10	264.80–825.0
Soriano et al.^16^	“SOR”	$C_{p,ion}\left(J\cdot {mol}^{-1}\cdot K^{-1}\right)=A+B\left(\frac{T}{K}\right)+C{\left(\frac{T}{K}\right)}^{2}$	10	14		188.06–663.10	264.80–739.0
Farahani et al.^22^	“FAR”^b^	*C*_p_ (J·mol^–1^·K^–1^) = −122.16826 + 0.45794*T* + 12.8395N_cation_ – 56.85424CH_3_R_cation_ + 19.25836N_anion_ – 11.36109*n*H_anion_				188.06–663.10	264.41–1396.96
Sattari et al.^21^	“SAT”	*C*_p_ (J·mol^–1^·K^–1^) = *A* + *BT* + *CT* ^2^	7	9		180.06–663.00	259.09–1805.7
Albert–Müller^18^	“ALM”^c^	*C*_p_ (J·mol^–1^·K^–1^) = *A* + *BT* + *CT* ^2^				190.00–663.10	268.70–1800.0
Müller–Albert^17^	“MIA”	$C_{p}\left(J\cdot {mol}^{-1}\cdot K^{-1}\right)=\sum_{i=cations}x_{i}C_{P,i}+\sum_{j=anions}x_{j}C_{P,j}$	39	32		190.00–663.10	268.70–1800.0
Ahmadi et al.^20^	“AHM”^d^	*C*_p_ (J·mol^–1^·K^–1^) = *a*_1_ *T*_2_^a^ + *a*_3_ ln(*T*) + a_4_MW_5_^a^ *a*_6_C_A_ + *a*_7_C_C_ + *a*_8_N + *a*_9_S + *a*_10_O + *a*_11_(F + Br + Cl) + *a*_12_B + *a*_13_				190.00–663.10	93.0–1764.0
Chen et al.^19^	“CHE”	$C_{p}\left(J\cdot {mol}^{-1}\cdot K^{-1}\right)=R\left[A+B\left(\frac{T}{100}\right)+D{\left(\frac{T}{100}\right)}^{2}\right]$	6	14	3	189.66–524.87	

a Empty fields indicate that the heat capacity range was not reported in the original publication.

b Farahani et al.’s method does not rely on predefined cation cores, anion cores, or substituent groups. Instead, the structural contributions enter the correlation directly through four descriptors (N_cation_, N_anion_, *n*H_anion_, and CH_3_R_cation_).

c The original paper does not explicitly report the exact numbers of cation groups and anion groups. Instead, the model comprises 36 cation/anion structural groups and 7 correction groups.

d In contrast to conventional group contribution approaches, this method is based on atomic contributions. It incorporates the number of individual atoms commonly found in ionic liquid structures, including the number of carbon atoms in the cation and in the anion as well as the number of nitrogen (N), sulfur (S), oxygen (O), phosphorus (P), fluorine (F), bromine (Br), chlorine (Cl), and boron (B) atoms.

All methods were originally developed to estimate the isobaric heat capacity of ILs at atmospheric pressure, which is consistent with the scope of this study. The table lists the temperature and heat capacity ranges used during model development and validation, as well as the main equation for each model, the numbers of cations, anions, and side groups proposed for each model in the application, and the prediction of the heat capacity of ionic liquids. Detailed descriptions of the models are provided in our Supporting Information (Supporting Information).

## 4 Evaluation of Group Contribution Methods

The accuracy of each model was scrutinized by comparing experimental and estimated data based on the following statistical parameters

1 $$\%\;RD_{i}=100\left(\frac{{C_{P}}_{i}^{\exp}-{C_{P}}_{i}^{calc}}{{C_{P}}_{i}^{\exp}}\right)$$

2 $$\%\;ARD_{i}=100\left|\frac{{C_{P}}_{i}^{\exp}-{C_{P}}_{i}^{calc}}{{C_{P}}_{i}^{\exp}}\right|$$

3 $$\%\;AARD=\frac{100}{N_{data}}\sum_{i=1}^{N_{data}}\left|\frac{{C_{P}}_{i}^{\exp}-{C_{P}}_{i}^{calc}}{{C_{P}}_{i}^{\exp}}\right|$$

where ${C_{P}}_{i}^{\exp}$ and ${C_{P}}_{i}^{calc}$ denote experimental and calculated heat capacities, respectively. The summation in eq 3 was carried out over all data points (*N*_data_) considered for a given analysis. It is important to note that *N*_data_ varied across models because some methods lacked the functional groups required to represent all ionic liquids occurring in our database. As a result, the application of these methods was not feasible in some instances. Additionally, the value of *N*_data_ depends on the level at which the statistical metrics are computed. In this work, *N*_data_ may correspond to (i) all data points associated with a single substance, (ii) all substances belonging to a specific cation family, or (iii) the entire set of ionic liquids included in the database.

Our evaluation procedure followed the same general strategy adopted in our previous study.^31^ In addition to the statistical analysis, we also examined the flexibility of the functional groups defined for each model. This qualitative criterion enabled us to assess whether the group definitions proposed in the original publications could adequately describe cations and anions that were not considered during model development. For this purpose, we carefully reviewed the fragmentation schemes underlying each of the methods. These aspects are further discussed in the next section.

## 5 Results and Discussion

Figure 1 illustrates the percentage of experimental data that each model could estimate. The figure shows that model coverage strongly depends on the chemical family of ionic liquids, with some models showing broad applicability. In contrast, others are restricted to a few cation–anion pairs. Among the eight methods evaluated, only the “FAR” and “SAT” models achieved full coverage of the database, meaning they were the only approaches capable of representing all ILs included in this study. This outcome is directly related to the fact that these models rely primarily on atom-based descriptors rather than fragment-based group definitions, which provide greater flexibility in describing diverse cation–anion combinations. In particular, the “FAR” model incorporates only four structural parameters: the total number of atoms in the cation and anion, the number of methyl groups in the cation, and the number of hydrogen atoms in the anion. Similarly, the “SAT” method combines atom counts with additional structural descriptors (e.g., the presence of triple bonds and heteroatoms) to enhance molecular characterization and broaden its applicability. As a result, both models could be applied to all ionic liquids examined in this work. The “AHM” model, although also based on atomic contributions, does not include the number of phosphorus atoms among its structural parameters and therefore cannot describe ionic liquids containing phosphonium-based cations.

**Figure 1:**
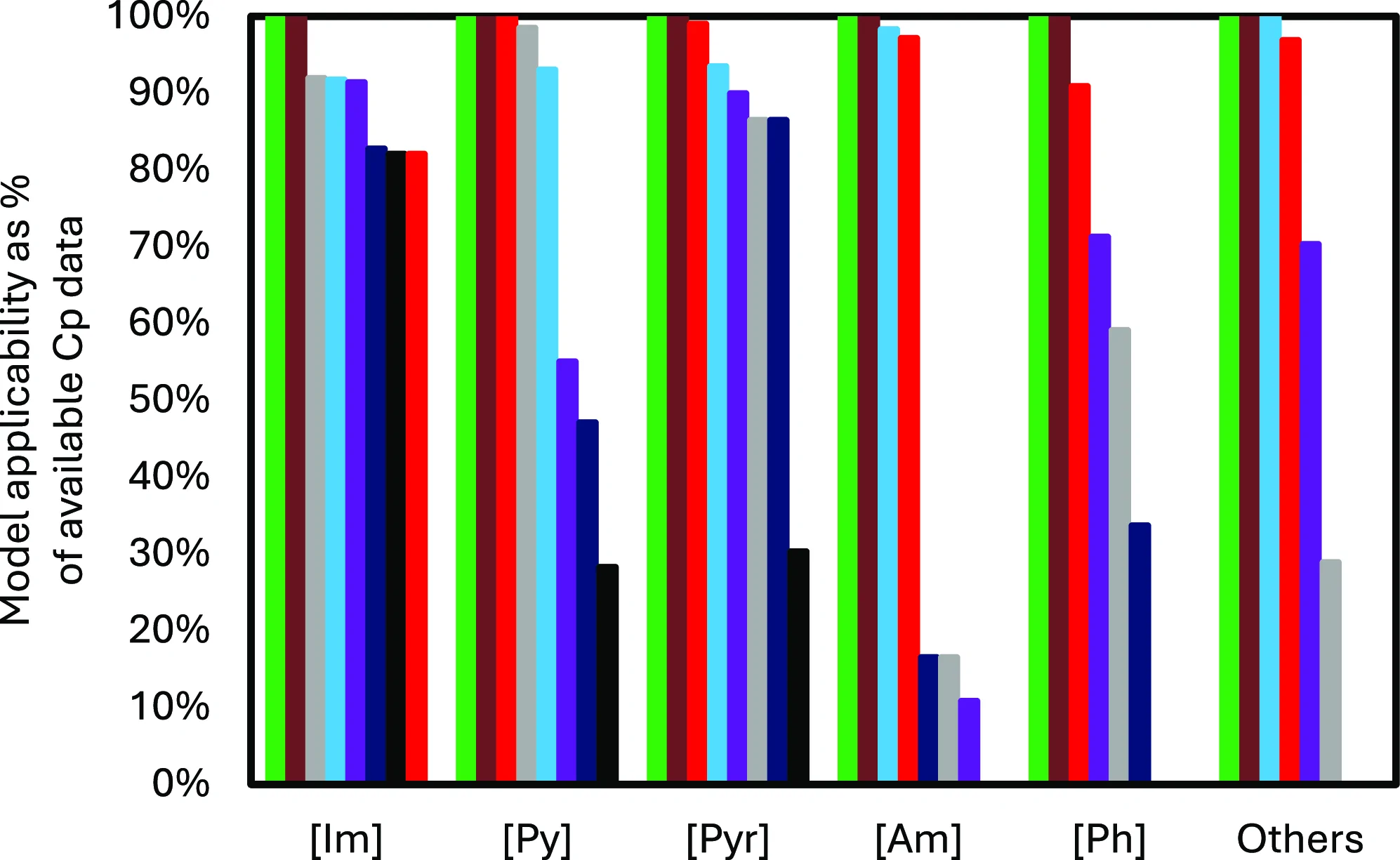
Percentage of *C*_p_ data estimated by each model: “FAR” model (green bar), “SAT” model (brown bar), “CHE” model (gray bar), “AHM” model (blue bar), “MIA” model (purple bar), “GAR” model (dark blue bar), “SOR” model (black bar), and “ALM” model (red bar). [Im] refers to imidazolium, [Py] refers to pyridinium, [Pyr] refers to pyrrolidinium, [Am] refers to ammonium, [Ph] refers to phosphonium, and [Others] include piperidinium, quinolinium, and isoquinolinium-based ILs.

The “SOR” method is restricted to imidazolium-, pyridinium-, and pyrrolidinium-based cations, which prevented its application to ionic liquids containing ammonium, phosphonium, or the cation types classified as “Others” in Figure 1 (e.g., piperidinium, quinolinium, and isoquinolinium derivatives). A similar limitation was observed for the “GAR” model, whose original set of structural groups was limited in its ability to represent pyridinium-, phosphonium-, and ammonium-based cations and their corresponding anions. The “CHE” model also exhibited limited applicability because it treats cations and anions as complete, undecomposed units (apart from additional CH_2_ and CH_3_ fragments) and thus does not account for side chains or other functional groups. As a result, it could not represent most ILs containing ammonium- or piperidinium-based cations, substantially restricting its applicability compared to other methods.

Table 2 summarizes the accuracy results for all models, organized into eight cation families. For each family, the table reports the % AARD values calculated for both the complete data set and the subset of data with experimental uncertainties ≤10%. In the complete data set, hyphens indicate cases in which a model could not be applied because the necessary functional groups were missing from its parametrization. In the subset with uncertainties ≤10%, hyphens may alternatively denote the absence of experimental data that meet that threshold, as is the case for phosphonium-, piperidinium-, quinolinium-, and isoquinolinium-based ionic liquids.

**Table 2:** Results of the Evaluation of Group Contribution Models for the Heat Capacity (*C*_p_) of Ionic Liquids in This Work^a^

Cation Family	N_IL_ ^b^	N_data_ ^c^	“GAR”	“SOR”	“FAR”	“SAT”	“ALM”	“MIA”	“AHM”	“CHE”
% AARD for all data points										
Imidazolium	65	6,830	2.94	4.29	13.97	7.11	29.60	5.08	6.73	17.51
Pyridinium	28	792	7.01	4.97	8.97	5.10	33.45	3.76	7.82	27.04
Pyrrolidinium	8	399	4.18	1.32	7.07	6.93	32.87	6.53	3.31	4.34
Ammonium	17	526	14.15	-	21.17	7.93	17.74	0.24	14.95	10.81
Phosphonium	20	362	3.27	-	16.30	17.59	11.39	2.21	-	17.52
Piperidinium	2	37	-	-	6.71	2.86	29.29	0.45	5.26	2.34
Quinolinium	1	77	-	-	23.87	27.42	36.74	0.06	34.36	-
Isoquinolinium	1	14	-	-	11.06	3.43	47.94	-	16.31	-
*N*_ILcationfamily_ ^d^			59	40	142	142	136	82	105	91
% AARD for data points with experimental uncertainties (NIST) ≤ 10%										
Imidazolium	42	3693	1.21	2.08	14.81	6.30	23.77	3.33	5.85	19.58
Pyridinium	12	194	2.73	5.94	9.85	4.74	40.37	6.10	8.15	43.54
Pyrrolidinium	3	91	3.06	-	2.61	5.30	24.08	8.71	2.42	2.46
Ammonium	5	140	8.49	-	36.81	2.82	25.19	0.03	9.75	3.12
Phosphonium^e^	0	0	-	-	-	-	-	-	-	-
Piperidinium^e^	0	0	-	-	-	-	-	-	-	-
Quinolinium^e^	0	0	-	-	-	-	-	-	-	-
Isoquinolinium^e^	0	0	-	-	-	-	-	-	-	-
*N*_ILcation family_ ^d^			31	28	62	62	61	42	53	47
**Overall** ≤ **10%**	**62**	**4118**	**1.92**	**2.36**	**15.04**	**5.67**	**27.17**	**3.97**	**6.41**	**24.25**
**Overall (all data)**	**142**	**9037**	**5.10**	**4.30**	**13.73**	**8.34**	**27.05**	**4.22**	**8.37**	**18.67**

a The values reported in the table correspond to the % AARD, as defined by eq 3. Fields marked with a hyphen indicate cases where the model could not be applied or where no data were available in the NIST database (particularly for the subset with uncertainties ≤10%).^26^

b Number of ILs available for this cation family.

c Number of experimental data points available for this cation family.

d Number of ILs whose data were estimated by this model.

e No heat capacity data were found in the literature for these cation families.

Considering the entire database, the ranking of model accuracy based on %AARD was “MIA” (4.22%) > “SOR” (4.30%) > “GAR” (5.10%) > “SAT” (8.34%) > “AHM” (8.37%) > “FAR” (13.73%) > “CHE” (18.67%) > “ALM” (27.05%). This ranking indicates that only a few models could provide accurate predictions across the diversity of ILs evaluated in this work. In particular, the superior performance of the “MIA”, “SOR”, and “GAR” methods can be attributed to their comprehensive parametrization for the two dominant cation families in the database (imidazolium and pyridinium), which together account for 84.34% of all data points. For example, despite its limited applicability to several cation types, the “SOR” model performed well for imidazolium- and pyridinium-based ILs, which helps explain its relatively low overall deviation. Likewise, the “GAR” and “MIA” models demonstrated robust overall accuracy, reinforcing the importance of both the model scope and functional-group parametrization in achieving reliable *C*_p_ predictions for structurally diverse ionic liquids. In contrast, the “FAR”, “CHE”, and “ALM” methods exhibited limited predictive accuracy for most cation families, resulting in consistently high % AARD values across the whole database.

When considering only data points with experimental uncertainty ≤10%, the ranking of model accuracy shifts to “GAR” (1.92%) > “SOR” (2.36%) > “MIA” (3.97%) > “SAT” (5.67%) > “AHM” (6.41%) > “FAR” (15.04%) > “CHE” (24.25%) > “ALM” (27.17%). Notably, restricting the analysis to high-quality experimental data places the “GAR”, “SOR”, and “MIA” models among the top three performers in terms of accuracy, reversing their relative order compared to the results obtained from the overall database. This result underscores the robustness of these models and their improved performance in *C*_p_ estimation when evaluated under more rigorous data quality criteria.

Beyond the overall rankings, a closer examination of the results highlights important strengths and limitations of the evaluated models. For instance, despite its good performance for imidazolium- and pyridinium-based ILs, the “SOR” method could estimate the heat capacity of only 65.85% of the 142 ILs in the database, primarily because it lacks parameters for most cation families and for several relevant anions, including thiocyanate (SCN^–^), bis­(oxalate)­borate (BOB^–^), and tetrachloroferrate (FeCl_4_^–^). Except for the ammonium family, the “GAR” model predicted the heat capacities of most of the 59 ILs it was originally designed for, performing particularly well for systems containing the “bis­(trifluoromethylsulfonyl)­imide” (NTF_2_^–^) and “hexafluorophosphate” (PF_6_^–^) anions. In comparison, the “MIA” model successfully modeled 82 of the 142 ILs in the data set, achieving overall %AARD values of 4.22% for the complete set and 3.97% for those with experimental uncertainty ≤10%.

The “FAR” model showed limited accuracy for imidazolium-, ammonium-, phosphonium-, quinolinium-, and isoquinolinium-based ionic liquids, with % AARD values exceeding 10% (see Table 2). Overall, it produced a % AARD of 13.73%, and more than half of its predictions (57.04%) resulted in deviations greater than 10%. These findings suggest that although the atom-based formulation of the “FAR” model allows it to cover all ILs in the database, the use of only four structural descriptors does not appear sufficient to ensure accurate *C*_p_ predictions. Meanwhile, the atom-based “SAT” model performed acceptably for most cation families, despite its poor results for phosphonium-based ILs (% AARD = 17.59%). The improved performance of the “SAT” model relative to the “FAR” model may be attributed to the larger number of molecular descriptors included in its correlation (16 parameters for “SAT” compared to only 4 descriptors for “FAR”). The large deviations of the “AHM” model for ammonium-based ILs could be attributed to the absence of hydrogen atoms in its original parametrization. Ammonium ILs often contain many hydrogen atoms in their cation chains, such as in “tributylammonium bis­[(trifluoromethyl)­sulfonyl]­amide” and “triethyloctylammonium bis­[(trifluoromethyl)­sulfonyl]­imide”. The lack of parameters for hydrogen groups likely leads to notable errors and systematic under- or overestimation of heat capacities. A closer look at the “CHE” model shows consistently high % AARD values when predicting data for many ILs in our database. This model particularly struggles with ILs containing phosphorus atoms in the anion core. For ILs containing anions such as tetrafluoroborate (BF_4_^–^) and hexafluorophosphate (PF_6_^–^), all estimates resulted in a deviation greater than 30% (see Supporting Information).

The large deviations observed for BF_4_^–^ and PF_6_^–^ ionic liquids arise from limitations in the data set used by Chen et al.^19^ to regress the corresponding group parameters. Only 301 data points were available for BF4^–^, and although 1697 points were available for PF_6_^–^, 1528 of them correspond to a single IL (1-butyl-3-methylimidazolium hexafluorophosphate) and to temperatures above 300 K. This limited structural and temperature diversity leads to a less robust parameter estimation. Consequently, when the model is applied to a broader and chemically diverse data set, as in the present work, larger deviations naturally emerge. Since our database contains 21 ILs with BF_4_^–^ or PF_6_^–^ as their anion, these inaccuracies contribute significantly to the overall % AARD reported in Table 2.

Table 3 summarizes the accuracy results for all models, organized into 12 anion families. The evaluation of group contribution models organized by anion families confirms the trends observed in the cation-based analysis (Table 2). Overall, the “MIA”, “SOR”, and “GAR” models remain among the most accurate, exhibiting low % AARD for the most-represented anions in the databank, such as NTF_2_^–^, PF_6_^–^, and BF_4_^–^. However, small changes in the overall ranking emerge when the anion family is reorganized in the analysis. For example, while “MIA” and “SOR” performed well in the cation evaluation, the anion analysis shows particularly consistent performance by the “GAR” model across several families, including those with the largest number of experimental data. This subtle change in ranking suggests that the relative robustness of each model depends not only on the structural diversity of cations but also on the representativeness and quality of the data associated with each anion in the experimental set.

**Table 3:** Performance of Group Contribution Models for Predicting Ionic Liquid Heat Capacities, Organized by Anion Family^a^

Anion family	NIL^b^	Ndata^c^	“GAR”	“SOR”	“FAR”	“SAT”	“ALM”	“MIA”	“AHM”	“CHE”
% AARD for all data points										
NTf_2_^–^	32	2721	4.77	2.11	9.09	4.66	21.50	2.59	6.25	4.18
PF_6_^–^	12	2126	4.71	7.09	7.54	7.42	32.17	6.77	8.19	49.09
BF_4_^–^	10	842	7.50	4.24	12.05	5.42	34.03	4.75	4.27	55.97
CH_3_CH_2_SO_4_^–^	3	530	2.36	2.36	17.73	6.25	25.06	1.09	10.59	3.05
OTf^–^	7	494	2.80	3.17	13.52	4.03	29.66	4.38	3.87	2.61
Br^–^	9	381	9.4	4.72	19.83	6.85	39.3	7.05	17.44	7.09
DCA^–^	4	268	-	6.62	21.55	11.34	19.13	8.44	7.96	11.63
FAP^–^	5	149	-	-	3.42	5.19	12.94	1.36	-	-
CH_3_SO_4_^–^	5	109	-	8.97	15.75	8.47	31.15	5.09	7.49	26.38
Ac^–^	4	93	-	2.58	17.77	4.03	19.03	3.6	2.29	7.69
SCN^–^	3	88	-	-	22.51	7.2	31.97	11.63	11.39	-
Cl^–^	4	76	-	17.81	19.19	9.23	104.4	-	9.66	8.14
*N*_ILanionfamily_ ^d^			50	34	98	98	96	60	77	77
% AARD for data points with experimental uncertainties (NIST) ≤ 10%										
NTf_2_^–^	8	392	3.23	0.70	11.33	1.92	14.94	3.12	1.89	1.63
PF_6_^–^	10	1776	1.14	1.12	9.16	6.53	29.59	3.89	0.92	50.63
BF_4_^–^	8	659	2.95	2.92	12.30	3.08	28.81	4.74	1.1	58.47
CH_3_CH_2_SO_4_^–^	3	275	2.01	2.02	18.04	5.89	25.97	0.56	10.68	2.23
OTf^–^	3	92	2.84	3.75	10.41	6.85	21.19	8.07	2.55	5.92
Br^–^	7	337	1.36	1.22	27.30	3.23	39.68	5.38	16.31	3.01
DCA^–^	2	121	-	4.73	20.36	12.22	24.18	6.48	2.90	3.61
FAP^–^	-	-	-	-	-	-	-	-	-	-
CH_3_SO_4_^–^	3	39	-	6.06	12.85	6.45	36.88	6.02	7.74	31.78
Ac^–^	3	30	-	2.41	21.82	3.95	26.16	3.65	2.10	2.18
SCN^–^	1	20	-	-	29.15	9.85	17.49	1.05	13.19	-
Cl^–^	-	-	-	-	-	-	-	-	-	-
*N*_ILanionfamily_ ^d^			31	24	48	48	48	34	40	44
**Overall** ≤ **10%**	**48**	**3741**	**1.92**	**2.45**	**15.23**	**4.82**	**27.50**	**4.39**	**5.70**	**25.76**
**Overall (all data)**	**98**	**7877**	**5.41**	**4.70**	**12.47**	**5.98**	**29.45**	**4.56**	**7.55**	**19.83**

a The values reported in the table correspond to the % AARD, as defined by eq 3. Fields marked with a hyphen indicate cases where the model could not be applied or where no data were available in the NIST database.^26^

b Number of ILs available for this anion family.

c Number of experimental data points available for this anion family.

d Number of ILs whose data were estimated by this model.

Although the differences between the two evaluation approaches are not significant, the analysis by the anion family reveals some specific limitations of the models. The “CHE” model, for example, continues to perform poorly for phosphorus-containing anions, such as PF_6_^–^ and BF_4_^–^, directly reflecting the parametrization deficiencies discussed earlier. Similarly, the “FAR” and “ALM” models continue to show large deviations across most anion families, reinforcing that their low accuracy is not restricted to specific cation combinations but rather to structural limitations in their formulations. In contrast, the good performance of “GAR”, “SOR”, and “MIA” for the main anion families demonstrates that these methods have more balanced parametrizations between cations and anions, which may explain their stable ranking even when the analysis is performed from the anion perspective. Thus, the evaluation using anions complements the analysis using cations and confirms that the predictive capacity of the most robust models is relatively insensitive to the database’s structural distribution.

In addition to analyzing all cation–anion combinations, it is important to note that the model performance is also influenced by the representativeness of the structural groups in the database. Because some cations and anions appear in only a limited number of experimental data sets, the parameters associated with these groups were originally fitted using relatively little data, which may reduce their robustness. Consequently, for poorly represented cation–anion pairs, the models tend to exhibit a higher uncertainty and larger deviations. This behavior reflects the intrinsic dependence of group contribution methods on the availability and diversity of the experimental data. It should be considered when interpreting model performance for less common ionic liquid families and specific cation–anion combinations.

A related aspect concerns the physical interpretability and transferability of the GC parameters. Although the structural groups are chemically defined, the numerical values assigned to each group are empirical quantities obtained through statistical regression of the experimental data sets available at the time of model development. Thus, these parameters represent effective contributions calibrated within specific temperature domains rather than being fundamental thermodynamic quantities.

In this context, the present study provides a systematic assessment of the parameter transferability. By employing a substantially larger and more structurally diverse database than those originally used for model development, the GC approaches are evaluated beyond their initial calibration domains, enabling a realistic appraisal of how well the fitted group parameters extrapolate to new cation–anion combinations.

The versatility of functional group-based models and their ability to capture the structural diversity of cations and anions in the compiled database can be better understood by analyzing the fragmentation strategies adopted by each estimation method. Figure 2 illustrates this by decomposing the “1-butylpyridinium” cation, used here as a case study, and shows how each model partitions the molecular structure into functional or atomic groups based on distinct criteria. Variations in fragmentation strategies directly influence how many cation–anion combinations a model can accommodate and the extent to which its heat capacity predictions can be generalized. Models that employ more detailed or specific group definitions often exhibit greater flexibility in representing a wide range of IL families, but this may sometimes come at the cost of increased complexity or reduced predictive accuracy for underrepresented structures. Figure 2 highlights these methodological variations and underscores the importance of the fragmentation strategy for determining the reliability and applicability of *C*_p_ estimates across structurally diverse ionic liquids.

**Figure 2:**
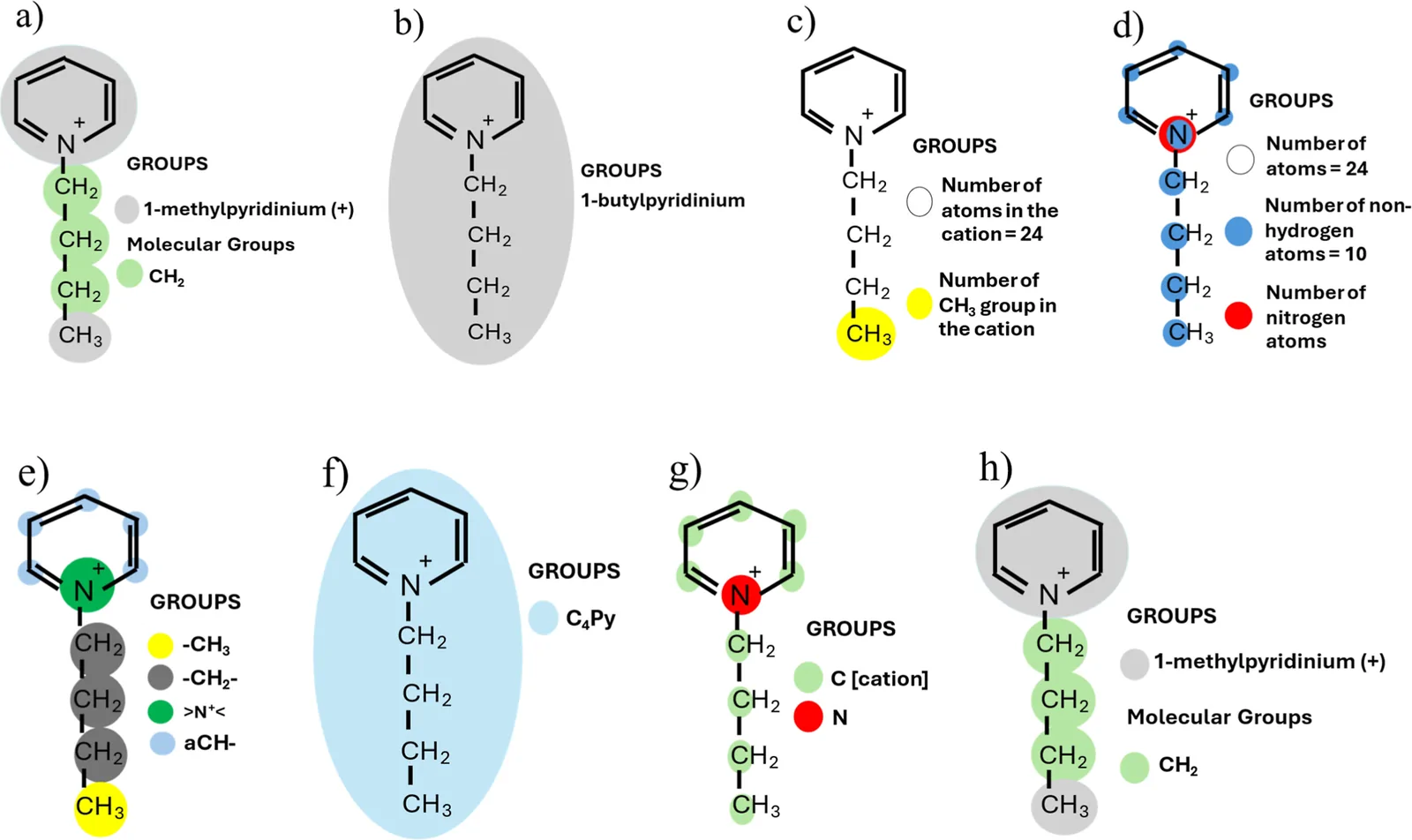
Fragmentation of the “1-butylpyridinium” cation into the groups of the following models: (a) Gardas and Coutinho,^15,32^ (b) Soriano et al.,^16^ (c) Farahani et al.,^22^ (d) Sattari et al.,^21^ (e) Albert and Müller,^18^ (f) Müller and Albert,^17^ (g) Ahmadi et al.,^20^ and (h) Chen et al.^19^

To further clarify the versatility and inherent limitations of the investigated models, a heatmap (Figure 3) was developed to illustrate the distribution of % AARD values across different cation cores and commonly reported anions. The heatmap should be interpreted as a measure of overall model coverage rather than as a direct comparison of the individual model accuracy. The objective is to delineate which cation–anion pairs are reliably captured by the evaluated models and to highlight those that remain challenging, often due to incomplete group parametrization or a lack of experimental reference data. An analysis of Figure 3 shows that the evaluated methods collectively demonstrate superior flexibility in describing ILs based on the imidazolium, pyridinium, and phosphonium cation families, which together account for 88% of the database examined in this study. It can also be seen that certain cation–anion combinations, such as imidazolium/chloride and pyridinium/sulfate, tend to yield higher % AARD values, suggesting potential limitations in how the models treat specific functional groups or interaction patterns.

**Figure 3:**
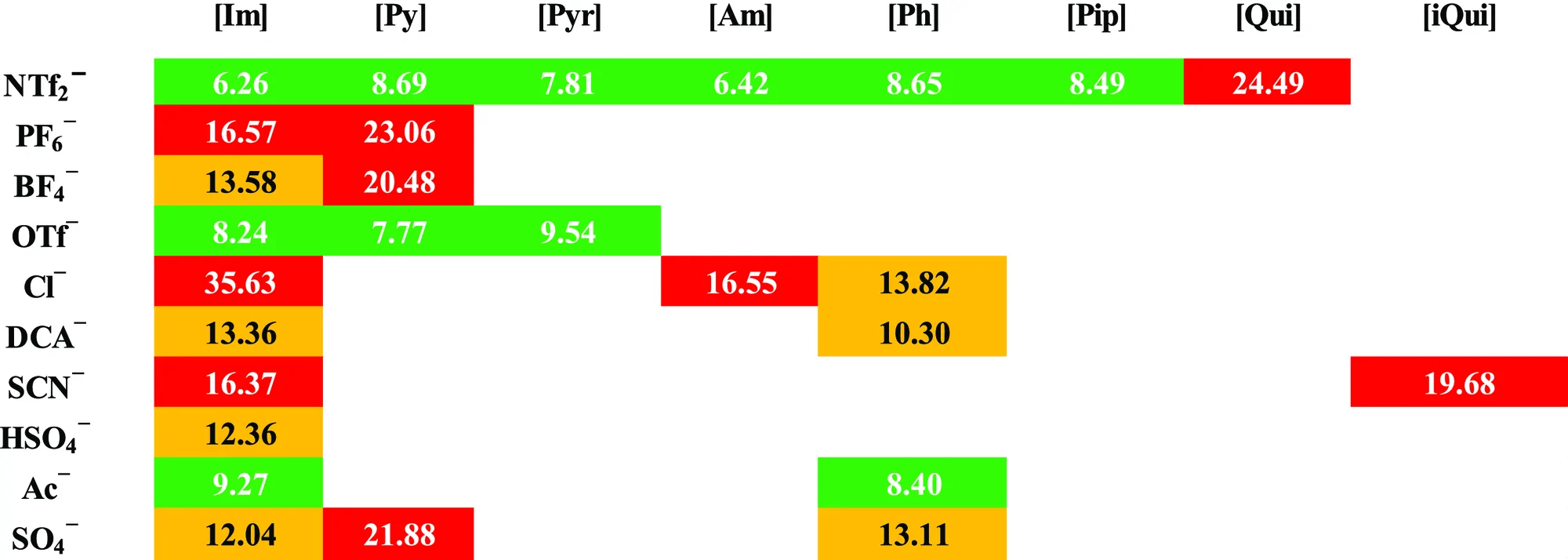
Heatmap of % AARD values for cation families and common anions. Colors indicate the level of deviation: green for % AARD <10%, yellow for 10% ≤ % AARD <15%, and red for % AARD ≥15%. Blank cells correspond to combinations not evaluated or not covered by the models.

In contrast, ILs comprising imidazolium/NTf_2_^–^ and pyridinium/OTf^–^ exhibit significantly lower deviations, indicating strong predictive performance for these extensively studied pairs. These trends highlight how the definition of functional groups, together with the amount and quality of experimental data, can significantly affect a model’s ability to predict heat capacity. Finally, the consistently high deviations associated with PF_6_^–^ and BF_4_^–^ arise mainly from the poor performance of the “CHE” model for these anions, which substantially increases their corresponding % AARD values.

Figure 4 shows the influence of temperature on the accuracy of the examined models. The observed deviations, both positive and negative, reflect overestimation and underestimation across the temperature range considered. Although no clear pattern emerges, it is notable that, except for the “FAR” (Figure 4c), “ALM” (Figure 4e), and “CHE” (Figure 4h) models, the deviations mainly occur within temperature ranges typical of industrial settings, specifically 273.15 to 373.15 K. Most *C*_p_ measurements are concentrated within this temperature range, especially for ILs that contain anions such as NTf_2_^–^, PF_6_^–^, BF_4_^–^, and alkylsulfate,^33–37^ which is also the range in which most group contribution parameters were originally regressed. As a result, most deviations naturally fall within this interval. Only a limited number of ILs have measurements extending beyond 400 K, and these high-temperature data points are unevenly distributed across anion families. This limited temperature coverage reduces the statistical influence of high-temperature deviations and limits the ability to detect potential trends.

**Figure 4:**
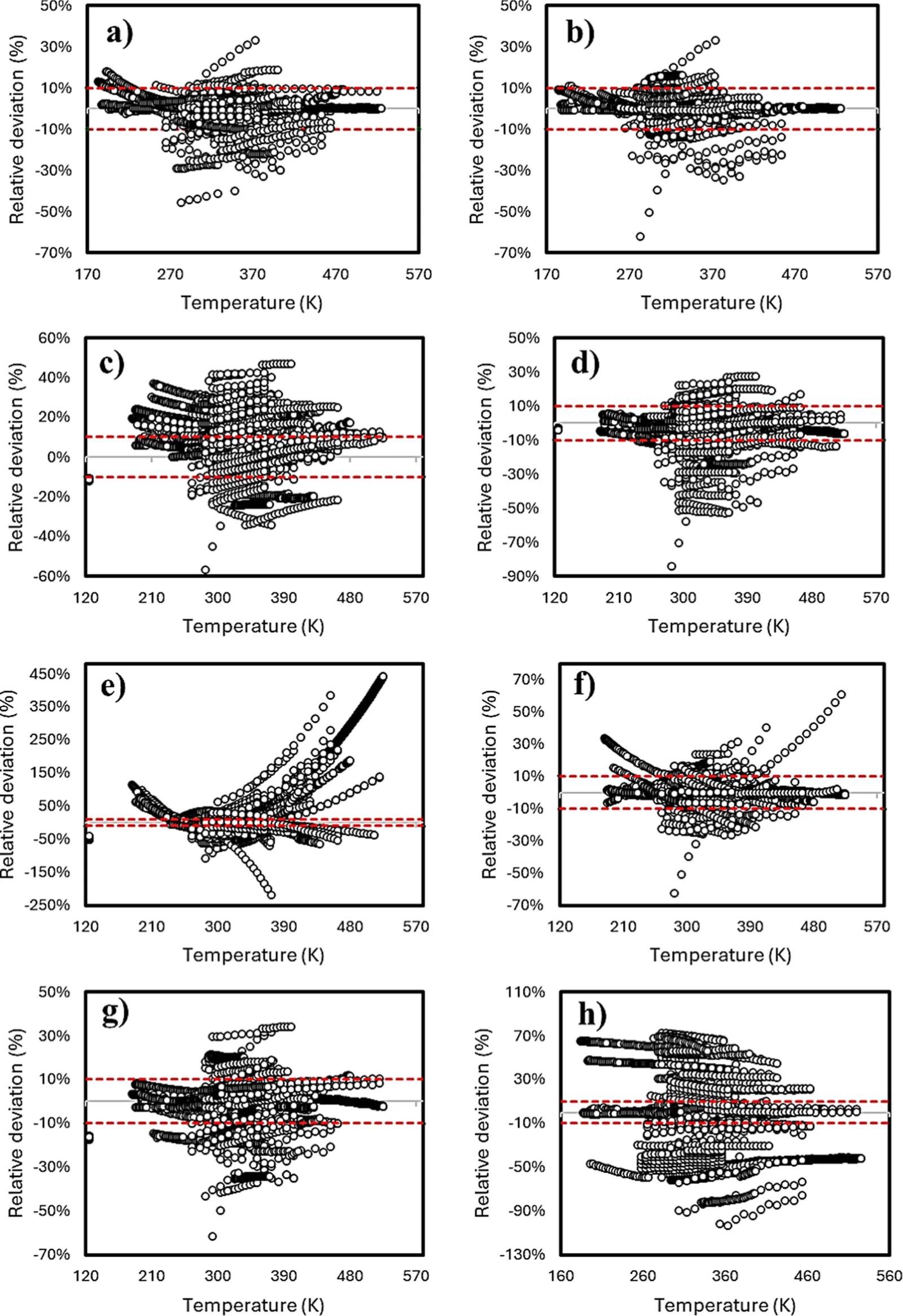
Relative deviations as a function of temperature calculated using the models of (a) Gardas and Coutinho,^15,32^ (b) Soriano et al.,^16^ (c) Farahani et al.,^22^ (d) Sattari et al.,^21^ (e) Albert and Müller,^18^ (f) Müller and Albert,^17^ (g) Ahmadi et al.,^20^ and (h) Chen et al.^19^

Another important factor influencing deviation patterns is the strong imbalance in the number of available data points across ionic liquid families. Our databank is predominantly composed of imidazolium- and pyridinium-based ILs, which together account for more than 7600 measurements due to their widespread industrial relevance and extensive characterization in the literature.^7^ In contrast, several other anion families, such as SCN^–^, Cl^–^, CH_3_SO_4_^–^, and BOB^–^, are represented by only a small number of ILs and by narrower temperature ranges. As a result, the overall %RD profile is primarily influenced by the chemical families well represented in the databank. In contrast, families with fewer data points have a much smaller statistical impact, even when their deviations are relatively large. This may explain why no clear temperature-dependent trend is observed in Figure 4: the distribution of deviations reflects not only the model’s performance but also the structural imbalance of the underlying experimental database.

In Figure 5, the relationship between estimated heat capacity and experimental values highlights clear trends in model behavior and inherent limitations. These patterns demonstrate that the models can accurately represent only certain regions of the chemical space for the ionic liquids tested while failing with cation–anion combinations or structures that differ from those originally included in model development. The occurrence of systematic deviations also indicates that some models may be overfit to specific chemical families, compromising their ability to make reliable predictions when they are applied to more diverse systems.

**Figure 5:**
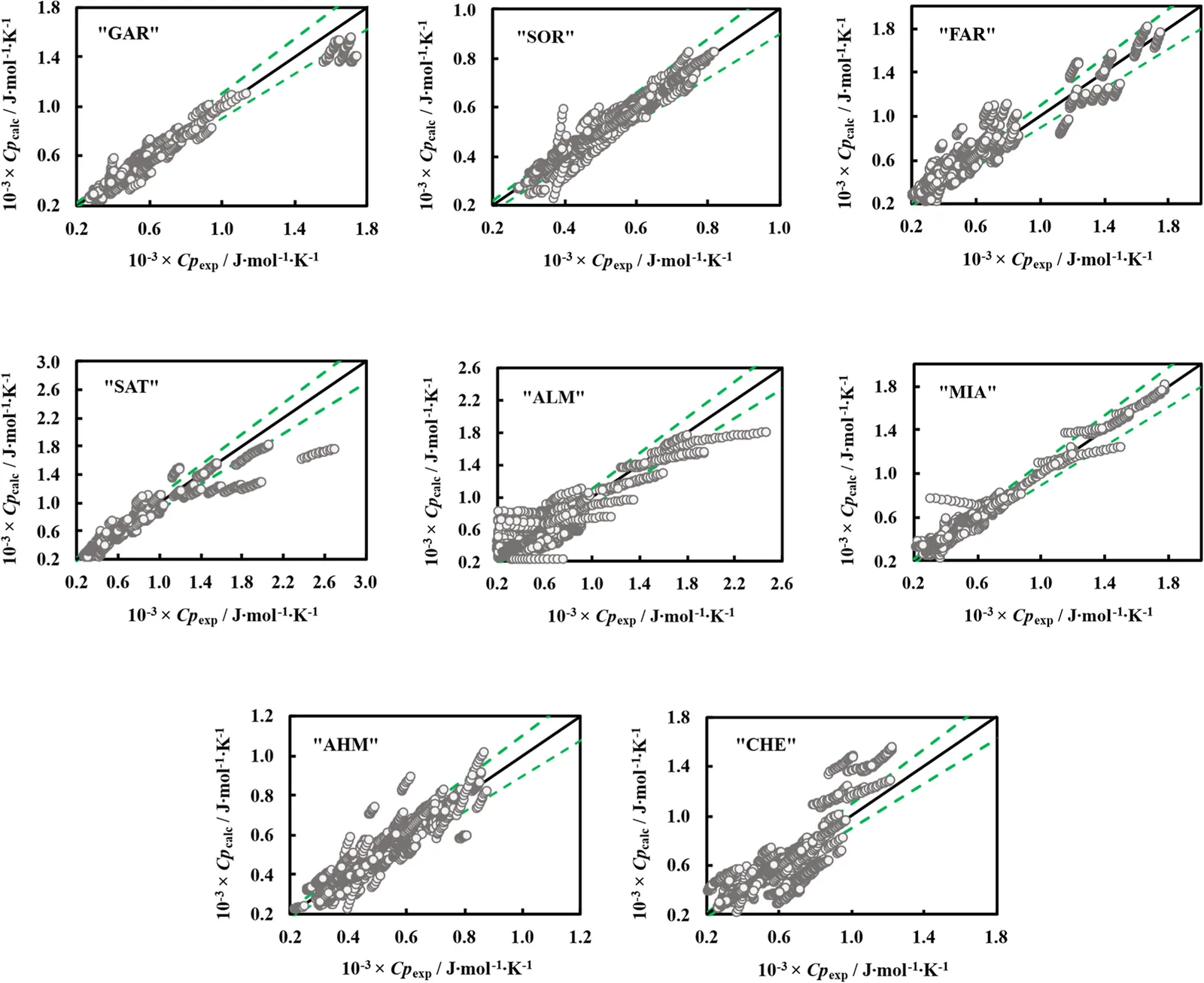
Estimated heat capacity versus experimental data for each method. Dashed lines correspond to ±10% relative deviation.

Furthermore, a comparison of the eight models reveals clear differences in the robustness and consistency of their predictions. Models such as “AHM” and “CHE” exhibit greater dispersion and more pronounced deviations, reflecting limitations in both the flexibility of their functional-group definitions and their ability to capture the structural diversity of cations and anions represented in the database. This variability in performance suggests that some models may be suitable only for restricted applications, while others demonstrate potential for broader use in process engineering contexts.

The tendency of some models to systematically over- or underestimate heat capacities is further explained in Figure 6. The “FAR” model shows a strong bias toward positive deviations, often underestimating the heat capacity of ionic liquids. Similarly, the “ALM”, “MIA”, and “AHM” models also tend to produce positive deviations, but with less frequent underprediction. In contrast, the “GAR” and “SOR” models produce more balanced results, with roughly symmetrical deviations, suggesting that their predictions are just as likely to be over- or underestimated. Meanwhile, the “SAT” and “CHE” models tend to have more negative deviations, suggesting a consistent overestimation of the heat capacity values for the ionic liquids studied.

**Figure 6:**
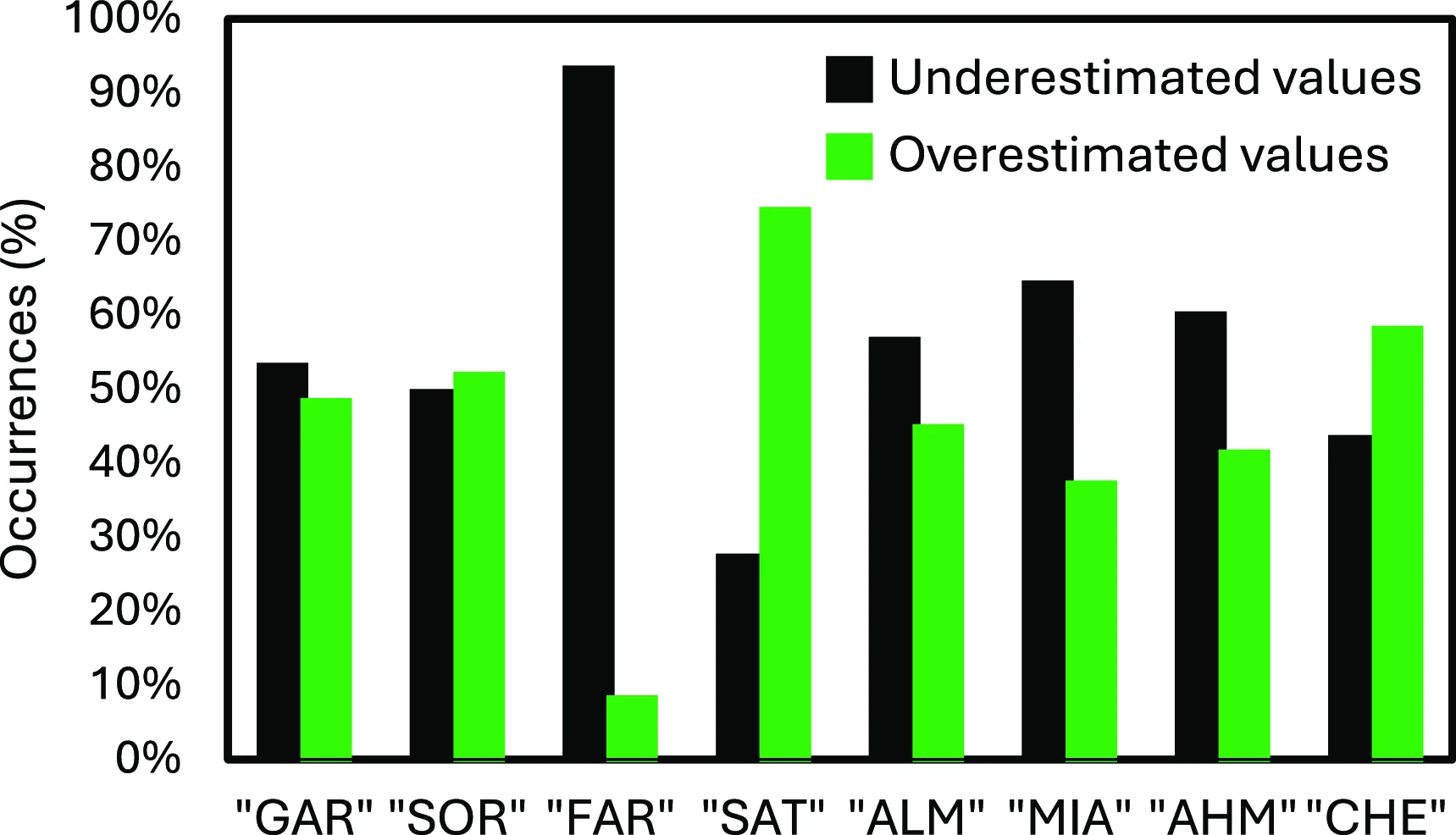
Occurrences of overestimations and underestimations for each model.

A closer molecular interpretation can also help explain why the evaluated GCMs exhibit such distinct levels of accuracy across the IL families. The superior performance of the “GAR”, “SOR”, and “MIA” models, for instance, can be attributed to how their functional group definitions capture the key molecular interactions influencing the heat capacity of ionic liquids (such as localized charge in aromatic rings,^38^ hydrogen-bonding patterns, and π-electron delocalization^2,39^ ). Imidazolium and pyridinium ions, which account for more than 80% of our database, possess well-characterized molecular interactions that are implicitly incorporated into the parametrization of the functional group definitions. As a result, models originally developed using extensive data sets of these aromatic cores, particularly imidazolium- and pyridinium-based ILs, inherently encode these molecular effects and tend to provide accurate predictions.

In contrast, nonaromatic cations such as ammonium, phosphonium, piperidinium, and pyrrolidinium exhibit distinct molecular characteristics (e.g., greater conformational flexibility and reduced charge delocalization), which can substantially influence the vibrational density of states^40,41^ and, consequently, the heat capacity of ionic liquids.^42^ These effects in nonaromatic ILs may cause deviations that cannot be corrected by group contributions originally fitted to aromatic systems. As a result, models with limited parametrization for nonaromatic cations, including ammonium, phosphonium, pyrrolidinium, and piperidinium, tend to underperform because these ions inherently affect the vibrational density of states and thus the heat capacity of ILs. The deviations observed in the scatter plots (Figure 5) and in the % AARD rankings are therefore not merely statistical; they reflect the extent to which each model succeeds or fails to represent the underlying molecular aspects governing heat capacity.

To further evaluate the performance of the investigated models, we explored how the deviations produced by each model are distributed across specific % ARD intervals to better demonstrate their overall accuracy and robustness. Figure 7 shows the distribution of average relative deviations (% ARD) across the entire database, which includes 9037 experimental data points. The analysis indicates that the “GAR” and “SOR” models achieve the highest accuracy, with 51.16% (3366 of 6580 data points) and 57.22% (3405 of 5951 data points), respectively, falling within the [0.0–1.0%] %ARD range. In contrast, the “FAR”, “ALM”, and “CHE” models show significantly poorer performance, as evidenced by the large share of predictions exceeding 10% deviation: “FAR” with 66.87% (6043 of 9037 data points), “ALM” with 71.35% (6303 of 8834 data points), and “CHE” with 44.94% (3479 of 7742 data points) above the 10.0% threshold.

**Figure 7:**
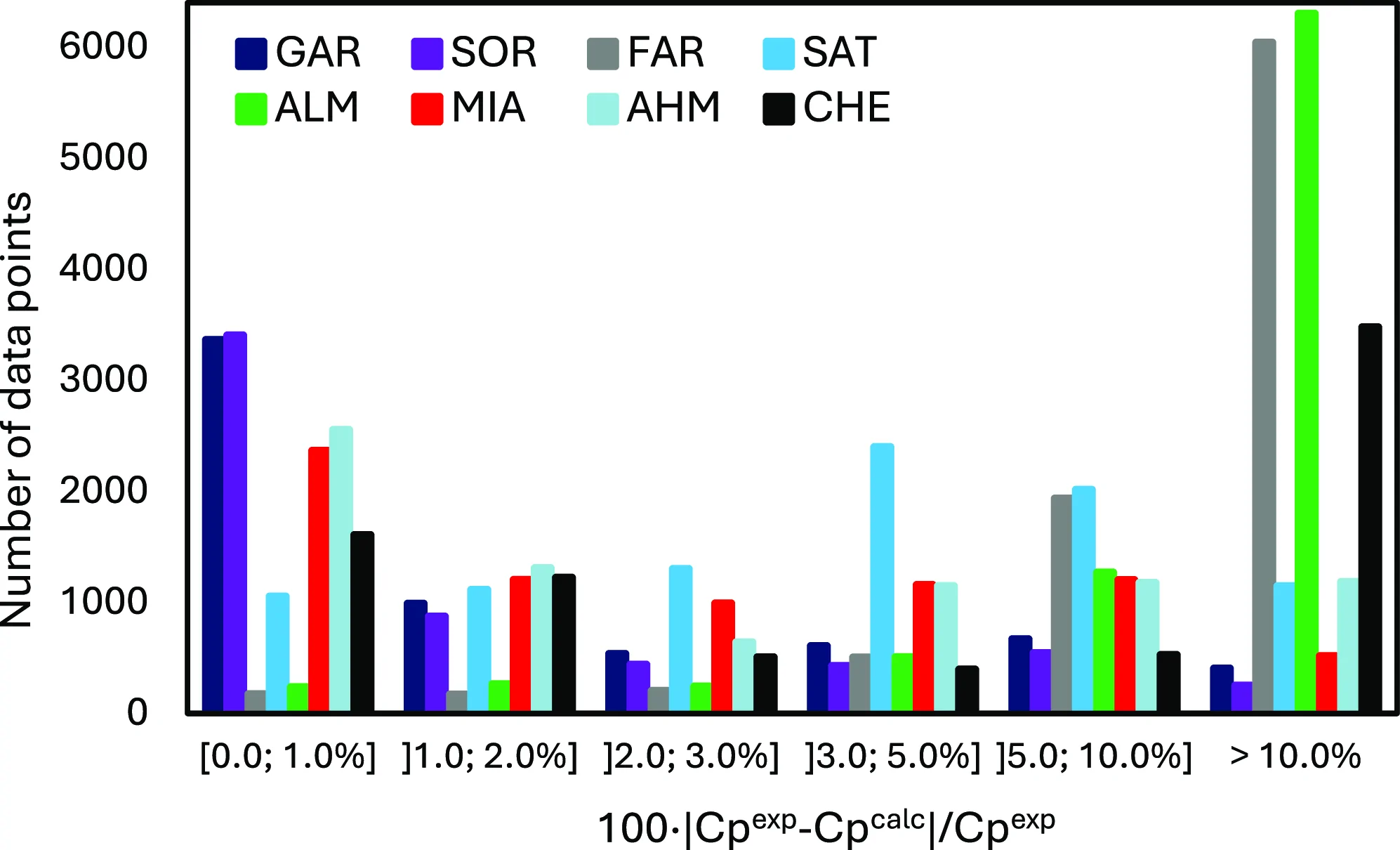
% ARD distribution for all models analyzed in this study.

These results reinforce the superior reliability of the “GAR”, “MIA”, and “SOR” methods while clearly exposing the limitations of some of the other models. Based on these results, the models were ranked according to the percentage of predictions within the reliable [0.0–5.0%] %ARD interval, as summarized in Figure 7: “SOR” (86.59%) > “GAR” (83.65%) > “MIA” (76.87%) > “AHM” (70.54%) > “SAT” (64.99%) > “CHE” (48.24%) > “ALM” (14.26%) > “FAR” (11.70%). This ranking clearly shows that models with a higher percentage of estimates within this accuracy window provide better predictions of ionic liquid heat capacities.^29^ Notably, both the “AHM” and “SAT” models, despite being atom-based methods rather than traditional group-contribution approaches, achieved strong performance within the [0.0–5.0%] interval, supporting their applicability to computer-aided molecular design^43^ (CAMD) in chemical engineering.

In the last part of our evaluation, an additional perspective on model performance was obtained by analyzing the average absolute relative deviations and the percentage of database coverage for each model (% AARD vs percentage of database coverage). Figure 8 illustrates the prediction accuracy (% AARD) and coverage of the evaluated GCMs.

**Figure 8:**
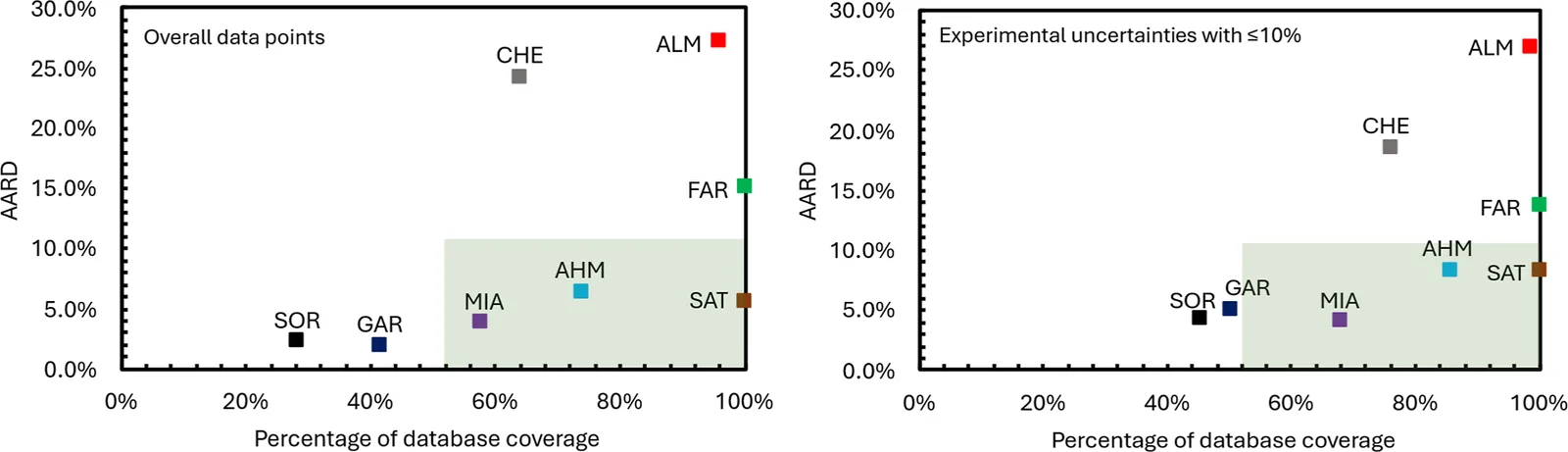
Comparison of the evaluated GCMs in terms of prediction accuracy (% AARD) and database coverage. Left: the complete database (9037 data points), and right: experimental data with reported uncertainties ≤10% (4118 data points).

As shown in Figure 8, a clear trade-off exists between accuracy and applicability for several of the evaluated models. The “SOR” model exhibits the lowest % AARD values in both databases, indicating good predictive performance. However, its applicability is restricted to a relatively small percentage of the database, limiting its practical use when a wide range of IL families must be considered. In contrast, “SAT” and “FAR” models are applicable to nearly the entire database, demonstrating significantly broader coverage while maintaining moderate % AARD values. Such characteristics make these models important alternatives for studies in chemical engineering, where, in some cases, a broader applicability may be more important than achieving the lowest possible deviations. In contrast, the “ALM” and “CHE” models exhibit comparatively high deviations despite their moderate flexibility to cover the database, indicating that greater coverage alone does not necessarily mean superior overall performance.

Therefore, the simultaneous consideration of prediction accuracy and database coverage provides a more realistic framework for selecting group contribution models for the estimation of ionic liquid heat capacities. From this perspective, models located toward the lower- right region of Figure 8 may be interpreted as the most balanced alternatives, as they combine broad applicability with acceptable predictive accuracy.

## 6 Conclusions

In this work, we systematically evaluated eight group contribution models to assess their ability to predict the temperature-dependent heat capacity (*C*_p_ vs *T*) behavior of ionic liquids. To achieve this, a comprehensive, curated database was created, including 9037 experimental *C*_p_ data points spanning a broad temperature range (190.00–524.87 K) for 142 ionic liquids. All available models were carefully evaluated based on their predictive accuracy, the extent to which their functional group parametrizations could represent the diverse structures of cations and anions in the data set, and their overall applicability, as outlined in the original references.

The comparative assessment clearly shows that the “GAR”, “SOR”, and “MIA” models have the highest percentage of predictions within the critical [0.0–5.0%] % ARD interval, demonstrating superior accuracy and robustness across the database. Notably, these three models also outperform the other models in terms of % AARD metrics, confirming their suitability for estimating the isobaric heat capacity of ionic liquids. Specifically, the “MIA” model is highly recommended for predicting the heat capacity of ionic liquids in the imidazolium and pyridinium cation families due to its lowest absolute average relative deviation (% AARD) compared with other models. Additionally, the “SAT” model is highlighted as a promising option for Computer-Aided Molecular Design (CAMD) applications, thanks to its flexible atomic-contribution framework and good % AARD performance for specific cation groups. Overall, the results presented here offer a solid foundation for selecting appropriate models in predictive tasks and design-oriented applications in chemical engineering.

## Supplementary Material



## Data Availability

Except for Chen et al.’s method, all calculations were carried out using the OCTOPUS v2.0 computational tool.

## Notes

Complete information about the validated data bank, including the registered ionic liquids, the experimental data points, and their literature references, and results output by all estimation methods (XLSX)

## ABBREVIATIONS

**Roman Letters**
GC: group contribution
CAMD: computer-aided molecular design
IL: ionic liquid
*T*: temperature
**Superscripts**
calc **=**: calculated
exp: experimental
